# Soil-transmitted helminths and *plasmodium falciparum* malaria among individuals living in different agroecosystems in two rural communities in the mount Cameroon area: a cross-sectional study

**DOI:** 10.1186/s40249-017-0266-6

**Published:** 2017-03-16

**Authors:** Irene Ule Ngole Sumbele, Gladys Belanka Nkemnji, Helen Kuokuo Kimbi

**Affiliations:** 1 Department of Zoology and Animal Physiology, University of Buea, Buea, Cameroon; 2 Department of Medical Laboratory Sciences, University of Bamenda, Bamenda, Cameroon

**Keywords:** Soil-transmitted helminths, *Plasmodium falciparum* malaria, Co-infection, Anaemia, Agroecosystem, Haematological values, Environmental contamination, Cameroon

## Abstract

**Background:**

Soil-transmitted helminths (STHs) and *Plasmodium falciparum* infections remain public health problems in Cameroon. A cross-sectional study was carried out in the Mount Cameroon area to determine the prevalence and intensity of STHs and *P. falciparum* infections in individuals living in different agroecosystems; to assess the influence of these infections on haematological parameters; and to identify the risk factors associated with STH infections.

**Methods:**

STH and malaria parasites were detected using the Kato-Katz method and Giemsa staining of blood films, respectively. Complete blood count values were obtained using an automatic haematology analyser. Soil samples were analysed using the sucrose floatation sedimentation method. Categorical and continuous variables were compared as required and logistic regression models were used to assess the risk factors for STH infections and anaemia.

**Results:**

Of the 450 participants examined, STHs, *P. falciparum* and mixed co-infections were detected in 14.0, 33.3 and 5.6% of participants, respectively. Significantly higher prevalences of *Ascaris* (18.8%) and *Trichuris* (7.9%) infections were observed in participants from tea plantation areas compared to those from banana and palm plantation areas, with similar trends in egg density. *P. falciparum* prevalence and parasite density were comparable between the different agroecosystems. The overall prevalence of anaemia was 64.2%. The prevalence of haematological manifestations such as moderate (48.0%) and severe (8.0%) anaemia, leucopenia (26.9%) and microcytosis (30.8%) was significantly higher among *Plasmodium-*STH co-infected participants. Soil samples from plantations showed the highest prevalences of STH eggs compared to soil samples from areas around pit toilets and public water taps. Living in a tea plantation area (*OR* = 3.07), age (A*OR* = 1.49) and lack of access to potable water (*OR* = 2.25) were identified as risk factors for STH infections, while the age groups 15–25 years (*OR* = 2.928) and 26–35 years (*OR* = 2.832), and being female (*OR* = 2.671) were significant risk factors for anaemia.

**Conclusions:**

STHs, malaria and anaemia are still of public health concern in plantation communities. Co-infections negatively influence haematological parameters. The tea farming agroecosystem, age and lack of access to potable water were identified as significant risk factors for STH infections.

**Trial registration:**

Not applicable.

**Electronic supplementary material:**

The online version of this article (doi:10.1186/s40249-017-0266-6) contains supplementary material, which is available to authorized users.

## Multilingual abstract

Please see Additional file 1 for translations of abstract into six official working languages of the United Nations.

## Background

Soil-transmitted helminth (STH) and *Plasmodium* infections remain a major health problem in many developing countries. Although STH infections are relatively preventable and easy to treat, some rural areas in Cameroon still record high prevalences of these infections [1, 2]. Closely associated with STH infections is malaria parasitaemia, with *Plasmodium falciparum* as the main species [3]. Malaria remains the primary reason for consultation in health facilities in Cameroon; it is responsible for 31% of consultations, 44% of hospitalisations as well as 18% of deaths occurring in health facilities in the country. In children below 5 years of age, 41% of deaths are due to malaria [4].

Several studies have shown that STH infections and malaria have remained endemic in many parts of the tropical world [5, 6] due to poverty [7], socio-economic problems and behavioural attributes [8]. Environmental factors such as the presence of bushes, stagnant water, poor sanitation, rainfall, low altitude and high temperatures may favour the growth and transmission of malaria parasites [9, 10]. Whilst farming represents an important livelihood strategy for a considerable number of rural and semi-urban dwellers, agricultural activities have also been associated with the transmission of both malaria parasites and STHs, especially hookworms [11, 12]. Previous studies have also shown that malaria-helminth co-infection patterns vary between different agroecosystems [13], hence the need to further investigate these co-infections in different agro-ecological settings. The lack of epidemiological data on STH and *Plasmodium* infections in rural farming communities hinders the development of informed policy on an already marginalised population.

Co-infections with STH and malaria may have considerable health consequences with controversial outcomes. While some studies have shown that helminths have a protective role [14, 15] others have associated helminth infections with adverse clinical outcomes in patients with malaria [16]. Although malaria and helminth infections are known etiological factors in tropical anaemia [17, 18], the extent to which their combined presence might interact to enhance the risk of other haematological abnormalities merits further investigation.

Previous studies in the Mount Cameroon area on malaria and helminth co-infection shave reported on their influence on the occurrence of anaemia, as well as the effect of altitude and urbanisation in children below 14 years of age [3, 19, 20]. The situation regarding older children, other age groups and farming communities in the area is unknown.

The government of Cameroon through the Ministry of Public Health has enhanced control measures against malaria and helminth infections. Such measures include the free distribution of long-lasting insecticide treated nets to all age groups, as well as free treatment of malaria among children below 5 years of age and the annual distribution of anthelmintics in primary schools since 2007 [21]. Following the intensification of control measures, more studies on polyparasitism in vulnerable populations are necessary to evaluate the impact of control efforts and to provide relevant information for policy-making.

Taking this into account, the present study was undertaken: to determine the prevalence and intensity of STHs and *P. falciparum* infections in individuals working in different agroecosystems in the Mount Cameroon area; to assess the influence of these infections on haematological parameters; and to identify the risk factors associated with STH infections. The findings of this study, especially for the group that is not targeted for control such as school-aged children not enrolled in schools and individuals older than 14 years of age, will provide valuable data to assist in the planning of control strategies in the country.

## Methods

### Study sites and participants

**Fig. 1:**
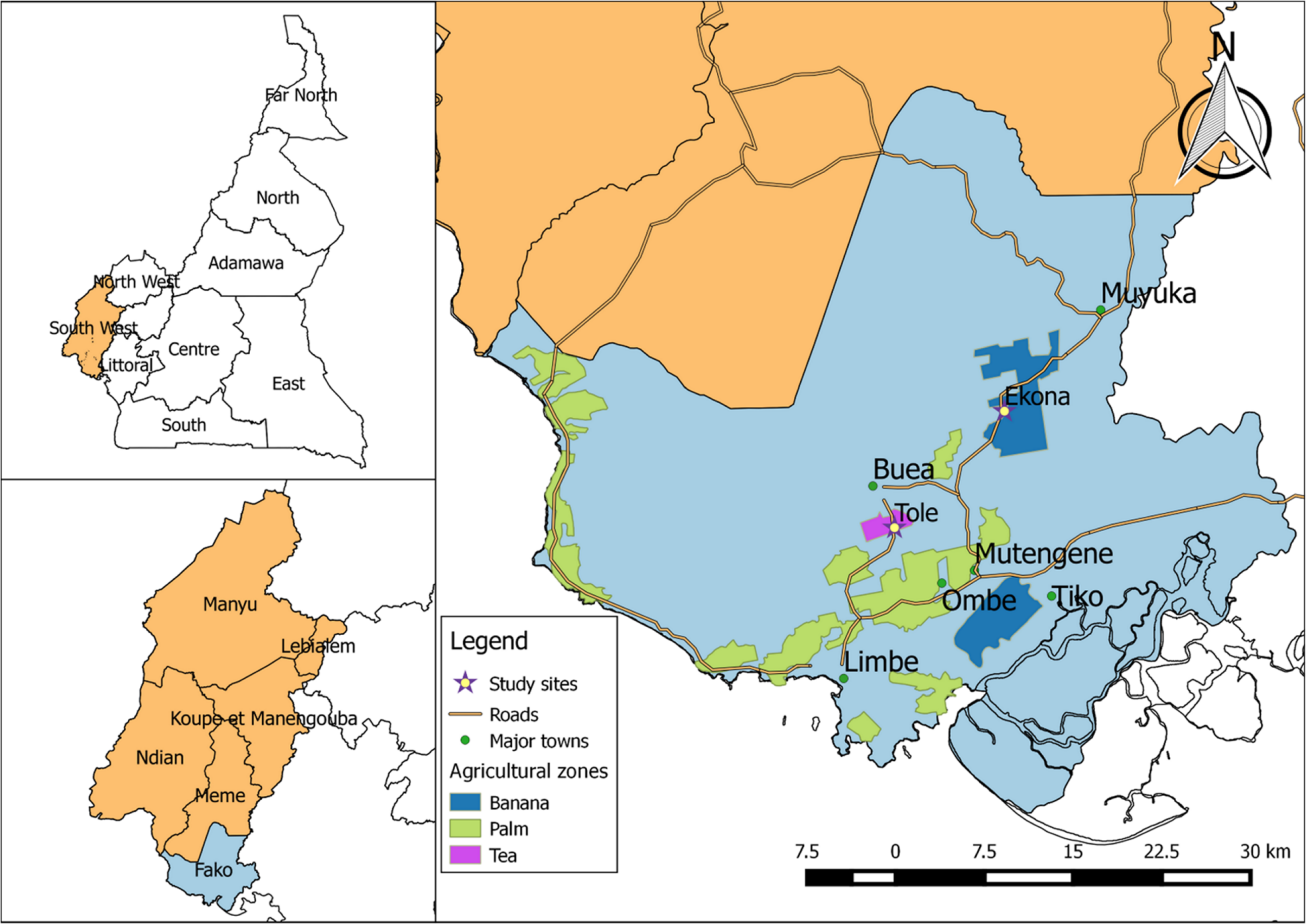
Map showing the location of the study areas in Fako Division, South West Region, Cameroon

This study was carried out in two rural farming communities in the villages of Ekona and Tole in Fako Division, South West Region. Both farming areas are situated in the Mount Cameroon area and have different agroecosystems, as shown in Fig. 1.

Mount Cameroon is a forested area, some of which has been cleared and replaced with banana, rubber, tea and palm plantations owned by the Cameroon Development Corporation (CDC) and small independent farm holdings. Banana and palm tree plantations are located in the southern part of the area, while tea plantations are located in the western part. The banana plantation is irrigated by the use of sprinklers and could be described as an environment of swampy flat land traversed by pools of irrigated water. The palm plantation is not irrigated and characterised by dense shade with little sunlight penetration. Tea plantations are characterised by short grass and shrubs that provide a wide range of pasture for livestock grazing.

The area’s climate is tropical, with temperatures ranging from 18 °C to 35 °C, and an average annual rainfall of about 4 000 mm [22]. Both Ekona and Tole have two distinct seasons: a cold rainy season from March to October, and a warm dry season with frequent light showers from November to February. The majority of inhabitants in the two villages are involved in farming, trading or livestock keeping. They are of poor socio-economic status, live in camp houses made of wood with holes and crevices, and have inadequate faecal disposal systems.

The study participants included individuals who farm, live and work in banana, palm or tea plantations. Members of their families who live in the workers’ camp with them between the ages of four and 60 years were eligible to take part in the study. Only individuals who willingly accepted to participate after being explained the purpose and protocol of the study, provided written or verbal informed consent (for adults) or assent (for minors), and have lived in the village for at least the past year were enrolled in the study.

### Study design

This cross-sectional study was carried out during the peak malaria transmission season between January and August 2014. The investigations entailed the use of a pre-tested, semi-structured questionnaire, measurement of body temperature using a clinical thermometer, and collection of blood, stool and soil samples for laboratory analyses. Due to a lack of information on exact population size of farmers in the different plantations, the sample size was calculated using the prevalence of *P. falciparum* malaria and helminth infections of a previous study: 64.2 and 38.3%, respectively, in the Mount Cameroon area [3]. The sample size was determined using the formula *n* = Z^2^pq/d^2^ [23], where *n* represented the sample size required; Z was 1.96, which is the standard normal deviate (for a 95% confidence interval, *CI*); p was 38.3%, the proportion of helminth prevalence; q was 1-p, the proportion of helminth negative; and d was 0.05, the acceptable error willing to be committed.

The optimum sample size was estimated to be 363. Considering a possible loss of samples due to blood clotting and non-consent to participating, the sample size was adjusted by 5% to a minimum of 381. A total of 485 eligible participants drawn by the lottery method using the monthly pay list of farmers working in the banana (140), palm (165) and tea (180) plantations were invited together with their children to take part in the study. Potential participants were contacted through the payroll officer on payday, and the research team and community health workers scheduled a day to explain the study’s purpose and protocol to the participants. Following this, potential participants were informed that a maximum of two persons, a parent and a school-aged child, would be recruited per family. The different localities for the daily collection of samples in the community centre hall were determined by the research team comprising a qualified clinician, a nurse and laboratory technicians. A total of 450 individuals consented to participate in the study, with a dropout rate of 7.2%.

Each individual’s body temperature was measured and individuals with a body temperature ≥ 37.5 °C were considered febrile. Parasitological and haematological analyses were performed on the blood samples collected to determine the presence of malaria parasites and to evaluate the blood elements. Collected stool samples were examined to determine the prevalence of STHs, while soil samples were examined to evaluate the environmental contamination with eggs or STH larvae in order to identify transmission hotspots.

### Questionnaire

Information on agricultural land use patterns, including crop types, storage and use of water in households and on agricultural plots was obtained from the participants using a pre-tested, semi-structured questionnaire. The questionnaire also elucidated participants’ socio-demographic information (age, sex, education level); use of malaria and helminth preventive measures (mosquito bed net use and wearing of shoes); pre-treatment of malaria and helminths; household conditions (block or plank house, floor type) and sanitation facilities (toilet facilities, access to potable water); and knowledge of and attitudes towards malaria and helminth infection transmission and control.

### Collection of blood, stool and soil samples

Approximately 4 ml of venous blood was collected from each consenting individual into sterile disposable syringes. Drops of whole blood were dispensed immediately on slides for the preparation of thick and thin blood films for the detection and speciation of malaria parasites, as described by Cheesbrough [24]. The remaining blood was dispensed into labelled ethylenediamine tetra-acetate tubes and placed in a cool box for transportation.

Clean, labelled, airtight plastic containers were given to each participant to return with a fresh midday stool sample. A single stool sample was collected from each participant.

Soil samples were collected within a 5-m radius of randomly selected anthropogenic activity sites in the community such as pit toilets, public water taps and yards of homes (surroundings), as well as the different plantations. Ten grams of topsoil was collected using a spoon to a depth of about 2 cm. The samples were collected in the morning and preserved in plastic bags. All collected samples were transported to the University of Buea Malaria Research Laboratory for analysis.

### Laboratory procedures

The air-dried thin blood film was fixed in 75% methanol, and both thick and thin blood films were stained using 10% Giemsa solution [24]. Slides were then microscopically examined for the presence of malaria parasites by two independent parasitologists, and in the case of any disparity they were read by a third parasitologist. Slides were considered positive when asexual forms and/or gametocytes of any *Plasmodium* species were observed on the blood film. Parasite density per μL of blood was determined on the basis of number of parasites per 200 leukocytes on thick blood film with reference to participants’ white blood cell (WBC) counts [24].

Asymptomatic malaria parasitaemia (AMP) was defined as the presence of *Plasmodium* parasites with an axillary temperature of < 37.5 °C, while clinical malaria parasitaemia (CMP) was defined as the presence of any species of *Plasmodium* together with an axillary temperature of ≥ 37.5 °C or reported fever in the previous 48 h, or headache or joint pain.

Full blood count was performed using a URIT-3300 Automated Hematology Analyzer (Guilin Botest Medical Electronic Co. Ltd, PR China), following the manufacturer’s instructions. Data on WBC, red blood cell (RBC) and platelet counts were obtained, as well as haemoglobin (Hb) level, haematocrit (Hct), mean corpuscular volume (MCV), mean corpuscular Hb and mean corpuscular Hb concentration. Individuals were considered anaemic when Hb concentration fell below the World Health Organization (WHO) reference values for age or gender [25]. Anaemia was classified as either mild (between 10.0 g/dL and the level in the WHO reference values for age or gender), moderate (7.0–10.0 g/dL) or severe (<7 g/dL) [24]. Leucopenia was defined as WBC < 4.5 × 10^9^/L, microcytosis as MCV less than73 fl [26] and thrombocytopenia was defined as platelet count < 150 000/μL.

Stool smears were prepared and examined using the Kato-Katz thick smear method, as described by Cheesbrough [24]. Duplicate smears were prepared for each specimen. Each slide was allowed to clear for 30 min, and then examined at 100× total magnification within one hour of preparation to avoid missing hookworm eggs. Morphological identifications of eggs of *A. lumbricoides*, *T. trichiura* and hookworm were based on identification aids, as described by Cheesbrough [24]. All the eggs in the 41.7 mg of stool were counted and multiplied by 24 to calculate the number of eggs per gram of faeces (epg). As a quality control measure, all positive slides and 10% of randomly selected negative smears were re-examined by a third experienced parasitologist who had no knowledge of the previous results. An average of the counts was utilised. For the STH species (*A. lumbricoides* and *T. trichiura*), the following egg count classifications were used to determine infection intensity: for *A. lumbricoides* infection, light = 1–4 999 epg, moderate = 5 000–9 999 epg and heavy = ≥ 10 000 epg; while for *T. trichiura*, light = 1–999 epg, moderate = 1 000–9 999 epg and heavy = ≥ 10 000 epg [27].

Approximately 2 g of sieved soil samples were analysed using the sucrose floatation sedimentation method, as described by Kagei [28]. For each soil sample, two independent analyses were performed, and if either of the two revealed eggs, then the sample was considered contaminated. STH eggs were identified with reference to the identification aids, as described by Cheesbrough [24].

### Statistical analysis

Analysis of the data was done using the IBM Statistical Package for Social Sciences (IBM SPSS Inc., Chicago, IL, USA) version 19. Data were summarised as means and standard deviations (SDs), and percentages were used in the evaluation of the descriptive statistics. Proportions were compared using the chi-square test (*χ* ^2^). Non-parametric tests such as Kruskal-Wallis test and Mann-Whitney *U* test were used to compare geometric mean egg density (GMED) of STHs across the different agroecosystems, sex and age groups (4–14, 15–25, 26–35, 36–45 and ≥ 46 years), as well as variations in mean haematological indices (Hb, Hct, RBC, WBC and platelet) with infection category where appropriate. Geometric mean parasite density (GMPD) of *P. falciparum* infections across the different agroecosystems and sex were compared using ANOVA and the Student’s *t*-test, respectively. Geometric means for STH eggs and *P. falciparum* counts were computed for those positive only and the log transformed STH eggs and *P. falciparum* counts were used in the analysis. Exploratory analyses were performed to select and prioritise variables to be entered into binary and nominal regression models. Any potential factor that had at least a modest (*P* < 0.2) relation with the outcome variable was included in the regression models. Binomial logistic regression (backward stepwise) models were run with the presence of any helminth as the outcome variable. In addition, nominal regression models were run with Hb concentration below the WHO reference values for age or gender as outcome variables to determine the risk factors for anaemia. All results were measured at 95% *CI*s and were considered to be significant at *P* < 0.05.

## Results

### Baseline characteristics

Out of the 485 eligible participants, a total of 450 (92.8%) individuals within the age range of 4–60 years (median age = 25.5 years) gave their consent or assent, and provided both faecal and blood samples for the evaluation of STH and *P. falciparum* infections, as well as haematological parameters. Of the 450 participants, 165 were tea plantation workers from Tole, while 127 were banana plantation workers and 158 were palm plantation workers from Ekona. The proportions of males (49.6%) and females (50.4%) in the study population were similar. A greater proportion of the participants belonged to the 4–14 years age group (37.6%) and the least belonged to the ≥ 46 years age group (9.1%), as shown in Table 1.

**Table 1:** Prevalence of fever, anaemia and (co-)infection status and parasite load of STH and *Plasmodium* infections, by agroecosystem, sex and age group

Parameter	Agroecosystem			Sex		Age group in years					Total	*P*-value		
	Tea % (*n*)	Banana % (*n*)	Palm % (*n*)	Male % (*n*)	Female % (*n*)	4–14 % (*n*)	15–25 % (*n*)	26–35 % (*n*)	36–45 % (*n*)	≥46 % (*n*)		*P* ^a^	*P* ^b^	*P* ^c^
Participants	36.7 (165)	28.2 (127)	35.1 (158)	49.6 (223)	50.4 (227)	37.6 (169)	12.4 (56)	21.3 (96)	19.6 (88)	9.1 (41)	450			
Fever	35.2 (58)	34.6 (44)	32.9 (52)	35.0 (78)	33.5 (76)	34.9 (59)	35.7 (20)	36.5 (35)	36.4 (32)	19.5 (8)	34.2 (154)	0.91	0.74	0.35
Anaemia	63.6 (105)	60.6 (77)	67.7 (107)	54.3 (121)	74.0 (168)	78.1 (132)	51.8 (29)	49.0 (47)	58.0 (51)	73.2 (30)	64.2 (289)	0.45	**<0.001**	**<0.001**
*Ascaris*	18.8 (31)	10.2 (13)	6.3 (10)	9.0 (20)	15.0 (34)	11.2 (19)	8.9 (5)	10.4 (10)	13.6 (12)	19.5 (8)	12.0 (54)	**0.002**	0.05	0.52
*A.l* GMED^Л^	1 550	1 055	1 408	987	1 696	1 658	1 998	1 544	907	1 200	1 388	0.72	0.40	0.81
	(96–10 800)	(72–9 600)	(720–4 800)	(72–7 200)	(96–10 800)	(96–1 080)	(1 200–2 400)	(96–9 600)	(72–4 800)	(240–3 840)	(72–10 800)			
*Trichuris*	7.9 (13)	1.6 (2)	2.5 (4)	2.2 (5)	6.2 (14)	5.9 (10)	3.6 (2)	3.1 (3)	0.0 (0)	9.8 (4)	4.2 (19)	**0.001**	**0.038**	0.074
*T.t* GMED^Л^	105	152	316	126	142	152	416	82	–	92	138	0.10	0.96	0.38
	(24–960)	(96–240)	(144–1 200)	(48–240)	(24–1 200)	(144–1 200)	(144–1 200)	(24–240)		(48–144)	24–1 200)			
STH Pos.	21.2 (35)	11.0 (14)	8.9 (14)	10.8 (24)	17.2 (39)	14.2 (24)	12.5 (7)	11.5 (11)	13.6 (12)	22.0 (9)	14.0 (63)	**0.003**	0.05	0.59
GMED total^Л^	1 202	1 249	919	735	1 498	1 236	1 276	1 308	907	978	1 142	0.538	0.121	0.969
	(48–11 760)	(72–9 600)	(144–4 800)	(48–7 200)	(96–11 760)	(48–11 760)	(144–2 400)	(96–9 600)	(72–4 800)	(144–3 840)	(48–11 760)			
MP Pos.	30.3 (50)	33.1 (42)	36.7 (58)	35.4 (79)	31.3 (71)	47.3 (80)	41.1 (23)	22.9 (22)	19.3 (17)	19.5 (8)	33.3 (150)	0.47	0.35	**<0.001**
*P.f* GMPD/μL^Л^	681	493	706	698	565	930	381	417	398	442	631	0.61	0.50	0.07
	(45–149 860)	(40–137 816	(63–22 848)	(45–137 816)	(40–149 860)	(60–149 860)	(45–25 560)	(51–60 756)	(40–42 963)	(64–11 550)	40–149 860			
AMP	14.5 (24)	13.4 (17)	25.3 (40)	15.7 (35)	20.3 (46)	27.2 (46)	23.2 (13)	12.5 (12)	5.7 (5)	12.2 (5)	18.0 (81)	**0.01**	0.21	**<0.001**
CMP	15.8 (26)	19.7 (25)	11.4 (18)	19.7 (44)	11.0 (25)	20.1 (34)	17.9 (10)	10.4 (10)	13.6 (12)	7.3 (3)	15.3 (69)	0.15	**0.01**	0.12
Co-infection	6.7 (11)	4.7 (6)	5.1 (8)	4.9 (11)	6.2 (14)	5.9 (10)	7.1 (4)	4.2 (4)	4.5 (4)	7.3 (3)	5.6 (25)	0.73	0.57	0.90

*Abbreviations*: *A.l GMED A. lumbricoides* geometric mean egg density per gram of faeces, *MP Pos.* malaria parasite positive, *co-infection* infection with STH and *P. falciparum*, *P. f GMPD/ μL P. falciparum* geometric mean parasite density/ μL of blood, *STH Pos* soil-transmitted helminth positive (*A. lumbricoides/T. trichiura*), *T.t GMED T. trichiura* geometric mean egg density per gram of faeces

*P* ^a^, *P* ^b^ and *P* ^c^ are *P*-values of agroecosystems, sex and age groups, respectively

*P-*values in bold are statistically significant (*P* < 0.05)

^Л^ Mean values are expressed as geometric means (range)

Overall, the prevalence of STH infections in the studied population was 14.0% (*CI* = 11.1–17.5%). Individuals were infected with one of two species of STHs: *Ascaris lumbricoides* (12.0%, *CI* = 9.3–15.3%) and *Trichuris trichiura* (4.2%, *CI* = 2.7–6.5%); no hookworm infections were detected. Out of the 63 individuals with STH infections, 54 (85.7%, *CI* = 75.0–92.3%) were infected with *A. lumbricoides*. The overall prevalence rates of light, moderate and heavy *Ascaris* infections were 87.0% (*CI* = 75.6–93.6%), 11.1% (*CI* = 5.2–22.2%) and 1.9% (*CI* = 0.3–9.8%), respectively. All *Trichuris* infections were light.

*P. falciparum* (33.3%, *CI* = 29.1–37.8%) was the only species of malaria ﻿parasite detected. Fever occurred in 34.2% (*CI* = 30.0–38.7%) of the participants, anaemia in 64.2% (*CI* = 59.7–68.5%) and CMP in 15.3% (*CI* = 12.3–19.0%) (see Table 1).

Mixed STH and *P. falciparum* co-infections were detected in 5.6% (*CI* = 3.8–8.1%) of the participants. The prevalences of *Ascaris*, *Trichuris* and *Ascaris* plus *Trichuris* were comparable in individuals with *P. falciparum* (13.3, 4.7 and 16.7%, respectively) and those without (11.7, 4.0 and 12.7%, respectively). Although not significant (*P* = 0.62), the GMED per gram of faeces of *Ascaris* was higher in malaria-negative individuals (1 522, range = 96–10 800) than malaria-positive individuals (1 187, range = 72–9 600). In contrast, the GMED of *Trichuris* was higher in malaria-positive individuals (196, range = 48–1 200) than in malaria-negative individuals (112, range = 24–960). Overall, the GMED per gram of faeces for individuals with STHs was higher among malaria-negative (1 325, range = 48–760) than malaria-positive individuals (909, range = 72–9 600).

### Influence of the agroecosystem

As shown in Table 1, the prevalence of STHs was significantly higher in participants from the tea plantation area (21.2%, *CI* = 15.7–28.1%) compared to the banana and palm plantation areas. Significantly higher prevalences of *A. lumbricoides* (18.8%, *CI* = 13.6–25.4%) and *T. trichiura* (7.9%, *CI* = 4.7–13.0%) were observed in the tea growing community as compared to the other agroecosystem communities. No significant differences were observed between agroecosystems and infection loads. Only one participant from the tea plantation area had a heavy infection (3.2%, *CI* = 0.6–16.2%).

For *P. falciparum,* the prevalence and densities were comparable in participants from the different agroecosystems. However, AMP prevalence was significantly higher in participants from the palm plantation area (25.3%, *CI* = 19.2–32.6%) as compared to those from the tea plantation (14.5%, *CI* = 10.0–20.7%) and banana plantation (13.4%, *CI* = 8.5–20.4%) areas. No significant differences were observed between the prevalence of anaemia and different agroecosystems (see Table 1).

### Effects of sex and age

STH infection prevalence and intensities were higher in females than in males. However, only the difference in prevalence of *T. trichiura* in females (6.2%, *CI* = 3.7–10.1%) and males (2.2%, *CI* = 1.0–5.1%) was statistically significant. No significant differences between age and prevalence of STHs were observed. Nonetheless, the prevalence of any STH was highest in participants of the ≥ 46 years age group than in their counterparts (see Table 1).

*P. falciparum* infection prevalence was comparable in males and females, however, it was significantly higher in participants aged 4–14 years (47.3%, *CI* = 40.0–54.8%) as compared to the other age groups. Although the GMPD/μL of blood was higher in males than in females and those aged 4–14 years, the differences were not significant. Overall, the prevalence of CMP was significantly higher in males (19.7%, *CI* = 15.0–25.5%), while AMP was significantly higher in participants aged 4–14 years (27.2%, *CI* = 21.1–34.4%) (see Table 1). Among the malaria-positive individuals, CMP was significantly higher (*χ* ^2^ = 6.32, *P* = 0.012) in males (55.7%) than in females (35.2%). In relation to age, CMP was highest in participants aged 36–45 years (70.6%) and lowest in those aged 46–60 years (37.5%) as compared to those aged 4–14 years (42.5%), 15–25 years (43.5%) and 26–35 years (45.5%). However, the differences were not significant (*χ* ^2^ = 4.83, *P* = 0.31).

Anaemia prevalence was significantly higher in females (74.0%, *CI* = 67.9–79.3%) than in males (54.3%, *CI* = 47.7–60.7%), and in participants aged 4–14 years (78.1%, *CI* = 71.3–83.7%) (see Table 1).

### Influence of infection on haematological parameters

As shown in Table 2, the prevalence of anaemia was significantly higher in individuals infected solely with *P. falciparum* (70.4%, *CI* = 61.9–7.7%) than in non-infected individuals (59.5%, *CI* = 53.5–65.3%). Likewise, anaemia prevalence was higher in those with co-infections (88.0%, CI = 70.0–95.8%) than in those with a single infection (67.7%, *CI* = 60.4–74.6%) and in those non-infected. Individuals with co-infections had a significantly higher prevalence of moderate (48.0%, *CI* = 30.0–66.5%) and severe anaemia (8.0%, *CI* = 2.2–25.0%) than their counterparts with single infections. Similarly, moderate and severe anaemia was significantly higher in participants with co-infections than in those non-infected.

**Table 2:** Prevalence and severity of anaemia, and mean haematological values as influenced by category of infection

Infection category	*n* and pairs	Anaemia	Anaemia severity % (*n*)			Mean (SD) values of:				
		% (*n*)	Mild	Moderate	Severe	Hb (g/dL)	Hct (%)	RBC (× 10^12^/L)	WBC (× 10^9^/L)	Platelet (× 10^9^/L)
*A.l* only	34	61.8 (21)	50.0 (17)	11.8 (4)	0.0 (0)	11.6 (1.4)	37.2 (4.3)	4.4 (0.4)	7.8 (3.3)	199.5 (81.5)
^a^ *T.t* only	12	50.0 (6)	33.3 (4)	16.7 (2)	0.0 (0)	11.4 (1.2)	36.9 (3.7)	4.3 (0.4)	7.8 (3.2)	248.3 (90.6)*
^b^Co-infection	9	55.6 (5)	33.3 (3)	22.2 (2)	0.0 (0)	11.1 (1.1)	35.9 (3.6)	4.2 (0.4)	7.5 (2.4)	246.9 (102.6)*
^c^ *P.f* only	125	70.4 (88)	46.4 (28)	23.2 (29)	0.8 (1)	11.1 (1.7)	34.4 (5.7)	4.2 (0.6)	8.9 (3.3)	187.3 (82.2)
^d^SI	162	67.7 (110)	46.3 (75)	21 (34)	0.6 (1)	11.2 (1.7)	35.0 (5.5)	4.2 (0.6)	8.7 (3.3)	191.7 (82.1)
^e^Co-infections	25	88.0 (22)	32.0 (8)	48.0 (12)	8.0 (2)	9.8 (1.9)	30.5 (5.9)	3.8 (0.7)	6.8 (2.9)	155.8 (68.9)
^f^Negative	262	59.5 (156)	45.0 (118)	14.1 (37)	0.4 (1)	11.8 (1.8)	36.9 (5.9)	4.4 (0.6)	8.4 (3.4)	189.2 (82.6)
Total	450	64.2 (289)	44.9 (202)	18.4 (83)	0.9 (4)	11.5 (1.8)	35.8 (6.0)	4.3 (0.6)	8.4 (3.3)	188.1 (81.9)
*P*-values of pairs of comparisons	^C^ vs ^f^	**0.039**	0.068			**<0.001**	**<0.001**	**0.010**	0.185	0.839
	^d^ vs ^e^	**0.011**	**0.001**			**<0.001**	**0.001**	**0.007**	**0.005**	0.065
	^d^ vs ^f^	0.084	0.183			**0.001**	**0.001**	0.059	0.224	0.965
	^e^ vs f	**0.001**	**<0.001**			**<0.001**	**<0.001**	**<0.001**	**0.019**	**0.049**

*Abbreviations*: *A.l Ascaris lumbricoides*, *P.f Plasmodium falciparum*, *SI* single infection, *T.t Trichuris trichiura*; vs: versus

^a^ *T.t* only = single infection with *T.t*

^b^Co-infection = infection with *A.l* and *T.t*

^c^ Single infection with *P.f*

^d^SI = infection with either*P.f*,*A.l* or *T.t*

^e^Co-infections = infection with *P.f* and *A.l* or *P. f* and *T.t or P.f*, *A.l* and *T.t*

^f^Negative = individuals negative for all infections

^a*^: Difference in mean platelet count for those infected with *T. trichiura* only and negative as determined by the Mann-Whitney *U* test (*P* = 0.01)

^b*:^ Difference in mean platelet count for those infected with *A. lumbricoides* plus *T. trichiura* and those negative by Mann-Whitney *U* test (*P* = 0. 047)

Comparisons of pairs of proportions of anaemia prevalence of ^C^ vs ^f^; ^d^ vs ^e^; ^d^ vs ^f^ and ^e^ vs ^f^ by *χ* ^2^

Comparisons of pairs of proportions of anaemia severity of ^C^ vs ^f^; ^d^ vs ^e^; ^d^ vs ^f^ and ^e^ vs ^f^ by *χ* ^2^

Comparison of pairs of mean haematological values of Hb, Hct, RBC, WBC and platelets counts of ^C^ vs ^f^; ^d^ vs ^e^; ^d^ vs ^f^ and ^e^ vs ^f^ by Mann-Whitney *U* test

*P-*values in bold are statistically significant

With regards to haematological values, individuals with co-infections had significantly lower mean Hb values [9.8 (1.9) g/dL] when compared to those infected with *P. falciparum* only, single infections and non-infected individuals. Similarly, lower mean values of Hct, RBC, WBC and platelet counts were observed in those with co-infections compared to their single infection or non-infected peers (see Table 2).

The significant variation in mean Hct and RBC values were comparable with the Hb values, with the exception of the mean RBC values, between those with single infections and those non-infected, which was not significant. Participants with co-infections had significantly lower mean WBC counts compared to their single infection counterparts and those non-infected, as shown in Table 2. Contrary to the variation observed in other haematological values, participants infected with *T. trichiura* only and co-infection with *A. lumbricoides* had significantly higher mean platelet counts [248.3 (90.6) × 10^9^/L and 246.9 (102.6) × 10^9^/L, respectively] than those non-infected. On the other hand, individuals with co-infections had significantly lower mean platelet counts [155.8 (68.9) × 10^9^/L] than their non-infected counterparts.

**Fig. 2:**
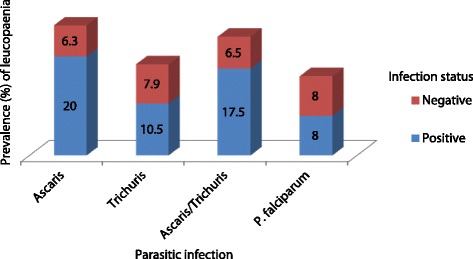
Prevalence of leucopenia as influenced by infection status and parasite species

**Fig. 3:**
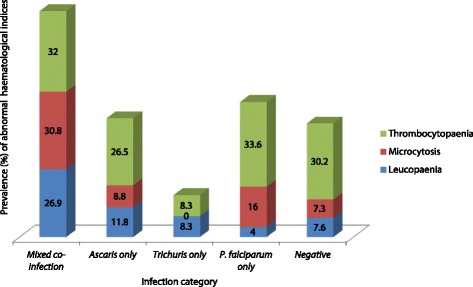
Prevalence of leucopenia, microcytosis and thrombocytopenia, by (co-)infection category

The prevalence of leucopenia, microcytosis and thrombocytopenia in the studied population was 8.0, 11.1 and 30.5% respectively. Overall, leucopenia was significantly higher in individuals just with *Ascaris* infection (*χ* ^2^ = 12.3, *P* < 0.001) and *Ascaris*-*Trichuris* co-infection (*χ* ^2^ = 8.9, *P* = 0.003) when compared with their non-infected counterparts (Fig. 2). The prevalence of microcytosis was significantly higher (*χ* ^2^ = 15.7, *P* < 0.001 and *χ* ^2^ = 7.15, *P* = 0.008, respectively) in individuals with co-infections (30.8%) and single infections with *P. falciparum* (16.0%). On the other hand, leucopenia was significantly higher (*χ* ^2^ = 14.0, *P* = 0.001) in those with co-infections (26.9%) than in those non-infected (see Fig. 3).

### Risk factors for anaemia

A multiple linear regression model with Hb level as the dependent variable revealed that sex (*P* = 0.001), malaria parasite density (*P* = 0.001) and WBC count significantly influenced Hb values (see Table 3). As shown in Table 4 significant risk factors associated with anaemia included being 15–25 years old (odds ratio, *OR* = 2.928), 26–35 years (*OR* = 2.832) and female (*OR* = 2.671), while participants with a normal body temperature were not at risk (*OR* = 0.622) of being anaemic.

**Table 3:** Multiple linear regression analysis exploring the relationship between Hb and independent variables

Parameter	B	SE	*t*-value	*P*-value
Sex	0.365	0.270	5.015	**<0.001**
Age	0.138	0.010	1.874	0.063
Malaria parasitaemia (log transformed)	−0.268	0.177	−3.375	**0.001**
Temperature	−0.044	0.191	−0.575	0.566
WBC	0.240	0.043	3.166	**0.002**

R^2^ = 0.256

*P*-values in bold are statistically significant

**Table 4:** Multinomial regression analysis examining factors associated with anaemia in the study population

Parameter	*OR*	95% *CI*	*P*-value
^a^Age group			
4–14 years	0.73	0.32–1.69	0.467
15–25 years	2.93	1.17–7.33	**0.022**
26–35 years	2.83	1.23–6.55	**0.015**
36–45 years	1.84	0.79–4.31	0.158
^b^Female	2.67	1.74–4.11	**<0.001**
^c^Malaria negative	0.76	0.45–1.25	0.272
^d^Helminth positive	0.78	0.37–1.65	0.514
^e^Co-infection	3.79	0.83–17.37	0.086
^f^Absence of fever	0.62	0.39–0.98	**0.043**
^g^WBC range			
Low (<4.5 × 10^9^/L)	0.96	0.19–4.94	0.960
Normal (4.5–16.4 × 10^9^/L)	1.372	0.33–5.69	0.662

^a^Age group: 4–14 years = 1; 15–25 years = 2; 26–35 years = 3; 36–45 years = 4; ≥ 46 years = 5

^b^Female =1; male = 0

^c^Malaria negative =1; malaria positive = 2

^d^Helminth positive = 1;helminth negative = 2

^e^Co-infection = 1; no co-infection = 2

^f^Absence of fever = 1; fever = 2

^g^WBC range low (<4.5 × 10^9^/L) = 1; normal (4.5–16.4 × 10^9^/L) = 2; high (≥16.4 × 10^9^/L) = 3

*P*-values in bold are statistically significant

### Risk factors for STH infections

Examination of soil samples collected from various locations in the communities in order to identify continuous transmission hotspots revealed that 25 (41.7%, *CI* = 30.0–54.3) of the 60 soil samples were contaminated with one or more STH species. *Ascaris* was the most common STH ova observed (36.7%) and no contamination with hookworm larva was observed. As shown in Table 5, the contamination of soils from specific sites in the communities was significantly different (*P* = 0.003). The highest prevalence of STH ova was found in soil samples obtained from plantations (73.3%) and the least prevalence of STH ova was found from around community taps (6.7%). The significant differences in prevalences of *Ascaris* (*P* = 0.018) and *Trichuris* (*P* = 0.039) in soil samples from the different sites followed the same trend.

**Table 5:** Prevalence of STH ova in soils from various sites in the communities

Factor		No. of samples examined	STH positive % (*n*)	*Ascaris* % (*n*)	*Trichuris* % (*n*)	*Ascaris* + *Trichuris* % (*n*)
Community	Tole	20	50.0 (10)	45.0 (9)	30.0 (6)	20.0 (4)
	Ekona	40	37.5 (15)	32.5 (13)	12.5 (5)	7.5 (3)
*P*-value			0.355	0.344	0.099	0.155
Sites	Pit toilets	15	46.7 (7)	47.6 (7)	13.2 (2)	13.2 (2)
	Taps	15	6.7 (1)	6.7 (1)	0.0 (0)	0.0 (0)
	Surroundings	15	40.0 (6)	33.3 (5)	20.0 (3)	13.2 (2)
	Plantations	15	73.3 (11)	60.0 (9)	40.0 (6)	20.0 (3)
Total		60	41.7 (25)	36.7 (22)	18.3 (11)	11.7 (7)
*P*-value			**0.003**	**0.018**	**0.039**	0.381

*P*-values in bold are statistically significant

The binomial logistic regression analysis revealed that agroecosystem more specifically the tea plantation agroecosystem (*OR* = 3.07), age (A*OR* = 1.49) and lack of access to potable water (*OR* = 2.25) were significantly associated with STH prevalence (see Tables 6 and 7). Participants working in the tea plantations were three times more likely to be infected with STHs, while those without access to potable water were two times more likely to be infected than their counterparts. Knowledge on helminth transmission and prevention and pre-treatment with anthelmintics showed no association with the prevalence of STHs.

**Table 6:** Unadjusted OR of factors associated with the prevalence of STH infections using the binary logistic regression model

Variable	*n*	STH prevalence % (*n*)	Unadjusted *OR*s (95% *CI*)	*P*-value
Agroecosystem				**0.007**
Tea	165	21.2 (35)	3.07 (1.51–6.22)	**0.002**
Banana	127	11.0 (14)	1.78 (0.79–4.04)	0.166
Palm	158	8.9 (14)	Reference	
Demographic information				
*Age groups*				
Age (years)				0.330
4–14	169	14.2 (24)	0.35 (0.09–1.32)	0.122
15–25	56	12.5 (7)	0.36 (0.09–1.38)	0.135
26–35	96	11.5 (11)	0.76 (0.25–2.35)	0.633
36–45	88	13.6 (12)	1.10 (0.36–3.35)	0.874
≥46	41	22.0 (9)		
*Gender*				
Female	227	17.2 (39)	1.40 (0.77–2.54)	0.269
Male	223	10.8 (24)	Reference	
*Education level of household head*				
Education level				0.485
Primary	277	12.6 (33)	0.52 (0.16–1.68)	0.274
Post-primary	150	14.0 (21)	0.66 (0.19–2.24)	0.505
*Malaria parasite status*				
Positive	150	16.7 (25)	1.38 (0.73–2.54)	0.302
Negative	300	12.7 (38)	Reference	
*Amenities and hygiene*				
Lack access to potable water	80	18.8 (15)	2.25 (1.29–3.91)	**0.004**
Access to potable water	370	13.0 (48)	Reference	
Seldom wearing shoes	12	33.3 (4)	0.30 (0.07–1.33)	0.114
Always wearing shoes	438	13.5 (59)	Reference	
Cemented house floor	432	14.1 (61)	0.36 (0.06–2.15)	0.264
House floor not cemented	18	11.1 (2)	Reference	

*P*-values in bold are statistically significant

**Table 7:** Final binary logistic regression model showing factors associated with the prevalence of STHs

Variable	*n*	Adjusted *OR*	95% *CI*	*P*-value
*Agroecosystem*				**0.002**
Tea	165	3.23	1.67–6.42	**0.001**
Banana	127	1.75	0.78–3.95	0.177
Palm	158	Reference		
* Age*	450	1.49	1.12–1.98	**0.007**
*Malaria parasite status*				
Positive	150	0.70	0.39–1.28	0.246
Negative	300	Reference		
*Amenities and hygiene*				
Lack access to potable water	80	2.20	1.35–3.58	**0.002**
Access to potable water				
Cemented house floor	432	0.55	0.12–2.65	0.459
House floor not cemented	18	Reference		

*P*-values in bold are statistically significant

## Discussion

Lack of effective interventions for and surveillance of neglected populations may lead to the resurgence and outbreaks of STHs and *P. falciparum* malaria. This study was designed to investigate the epidemiology of STH and *P. falciparum* infections in two rural communities with intensive agricultural land use practices, some of which have the potential to create favourable environments for the development of STH ova and suitable mosquito breeding sites. This scenario is likely to influence the transmission patterns of these infections, hence leading to unfavourable health conditions for the farmers and their families living in such communities [11, 29].

The overall prevalence of STHs in the study population of 14.0% is lower than the 38.3% reported in children in the Mount Cameroon area [3] and 43.8% in children in Ekona [2]. On the other hand, the observed prevalence is higher than the 2.5% reported in a recent study of various sites in the Mount Cameroon area [20]. The observed decrease in overall prevalence in this rural setting may be credited to the impact of helminth control strategies employed by the government. Current control strategies have focused on preventive chemotherapy through mass drug administration, in which at-risk populations such as school-age children and pregnant women are treated once or twice per year with benzimidazoles. While preventive chemotherapy can greatly reduce morbidity from helminth infections, re-infection may typically occur rapidly following treatment [30]. This is especially likely in children living in rural areas and farming communities with low levels of sanitation. In addition to the lack of adequate faecal disposal facilities, adults including farmers who are often left out of the national de-worming programme [21] may harbour a good number of these worms, hence serving as a main source of faecal contamination with helminth ova in their surroundings.

Only two of the three common STHs (*A. lumbricoides* and *T. trichiura*) were observed in the studied population. A similar observation was made by Zeukeng et al. [19] in Mfou Health District in Cameroon and Nkengazong et al. [1] in Marumba II who reported that hookworm was rare in the entire Mount Cameroon area. However, as most plantation workers are provided with personal protective working boots, hookworm infections that result from direct percutaneous invasion of infective larvae found in the soil did not have the opportunity to penetrate the skin, while *Ascaris* and *Trichuris* eggs which are transmitted faecal orally could easily be picked up. Furthermore, findings from this study revealed an absence of hookworm larvae in the soil samples collected from specific sites in the communities, even though a 41.7% contamination with geohelminth eggs was observed. The presence of eggs in the soil is indicative of faecal pollution corroborating the inadequacy of toilet facilities in the area. Hence, in addition to the provision of appropriate means of faecal disposal in rural communities, there is a need to incorporate behavioural change and health education in the control programme, if the desired goal of STH elimination is to be achieved.

The higher prevalence of STHs (*A. lumbricoides* and *T. trichiura*) infections in participants from the tea plantation area than in their counterparts from the banana and palm plantation areas is not unusual. Tea plantation workers were three times more likely to be infected with STHs than their counterparts. Elsewhere, higher prevalence of STHs than those observed in this study have been linked to tea farming communities [31]. The presence of favourable factors for the growth and transmission of STHs in the tea plantation communities could possibly be linked to the fact that the production of tea in Cameroon is not GLOBALGAP certified, while the production of banana and palm by the CDC is. GLOBALGAP is an internationally recognised set of farm standards dedicated to good agricultural practices, and certification assures adherence to the standards of health, safety and welfare of workers as well as the environment. Moreover, because tea is a shrub with a closed canopy, it may provide a suitable cover and inspire individuals to defecate more freely than in the open canopies of banana and palm plantations. Defecation practices in other tea growing communities have been reported as a strong risk factor for acquiring STH infections [31].

The widespread prevalence of STHs in more females than males is in line with studies carried out in Kumba, Cameroon and tea growing communities in India [31, 32]. On the other hand, studies in the same area [33] and in Mfou Health District [19] revealed the contrary. The higher prevalence and density of *Ascaris* and *Trichuris* infections may be related to the population at risk of infection in the different agroecosystem and gender-related roles. Even though the overall numbers of males and females examined in this study were similar, more women (55.8%) than men (44.2%) were working as tea harvesters in the plantation. Furthermore, girls have been reported to be more likely to commence working as tea pickers than boys who engage in more strenuous factory work [31].

Although the univariate analysis did not significantly associate any age group with STH prevalence, the multivariate analysis (see Table 5) identified age as a risk factor for STH infection. Several studies have been carried out among at-risk groups such as (pre)school age children and pregnant women in various locations [1, 3, 33, 34], however, fewer studies have examined the prevalence of STHs in other age groups at risk of infection. The prevalence of STHs was highest in the 46–60 years age group, which is in contrast to the highest prevalence observed in school-age children in rural communities of Mfou Health District [19]. In addition, out of the 63 individuals with STH infections in this study, the majority (61.9%) were ≥ 15 years of age. The prevalence of STHs in this age bracket may be attributed to this age group participating in agricultural activities such as hand picking of weeds, harvesting of tea leaves and palm seeds, and planting, as well as being linked to poor hygienic practices while working in the plantations, which are contaminated with helminth ova. Furthermore, these age groups are excluded from the government-sponsored national helminth control programme.

Individuals without access to potable water were two times more likely to be infected than their counterparts. Whilst performing farming activities such as harvesting or weeding, hands are likely to come into physical contact with contaminated soil. In addition, the majority of the soil samples collected from the plantations were contaminated with either *Ascaris* or *Trichuris* ova, indicating faecal pollution. In the absence of access to potable water, such individuals are likely to eat food without proper washing of their hands. Hence, for the control of helminths to be effective, there is a paramount need for an improvement of sanitary habits through education and provision of potable water accessible to all.

The overall prevalence of malaria (33.3%) is comparable to observations at various sites and in different populations in the Mount Cameroon area [20, 35, 36], but lower than the 77.2% observed in Mfou [19]. Worthy of note is the reported decrease in malaria parasite prevalence that has been credited to the intensification of malaria control measures in the Mount Cameroon area [35]. However, the diverse geo-ecological and climatic conditions of the Mount Cameroon area and other regions might influence malaria vector breeding and distribution as well as malaria parasite prevalence in different areas. Nonetheless, while the higher prevalence of malaria parasites in the youngest age group is in line with other studies [19, 36, 37], the highest proportion of CMP observed in participants aged 36–45 years is remarkable. This unusual finding merits further investigation, as acquired immunity to malaria is both exposure- and age-dependent, and adults are likely to have developed some degree of immunity as a result of repeated infections [38].

While the significant symptomatology of malaria parasite in males may be linked to the high parasite densities, on the contrary, a significant tendency of asymptomatic malaria was observed in participants with high malaria parasite density from the palm plantation area and children belonging to the 4–14 age group. Although high parasitaemia has been associated with an increase in disease severity, this is not always the case as parasites that circulate in peripheral blood do not always accurately reflect the number of parasites due to sequestration [39]. This notwithstanding, the presence of a greater number of asymptomatic infections in the studied population which may often go undetected and untreated thus resulting in a major source of gametocytes for local mosquito vectors [40] poses an unseen hurdle to the control efforts against malaria transmission in the area.

The prevalence of anaemia (64.2%) found in this study is significantly higher than the 42% reported by a cross-sectional study conducted in Mfou [19]. While the higher prevalence of anaemia observed in female participants may be credited to the higher physiological demands for iron [41] by those in the reproductive age group, especially menstruating women, the higher prevalence of anaemia in participants aged 4–14 years may be attributed to the occurrence of significantly high prevalence and density of malaria parasites. Furthermore, the multivariate analysis revealed that participants aged 15–25 years and 26–35 years are at a 2.8-fold risk of acquiring anaemia, which may be credited to the higher prevalence of CMP in these groups. In line with previous studies [42, 43], anaemia prevalence was significantly higher in individuals who were malaria-positive. Worthy of note also is that the highest prevalence of anaemia was observed in those with co-infections.

The prevalence of STH and *P. falciparum* co-infections in the population (5.6%) was lower (22.1%) than that reported in a study of the rural communities in Mfou [19]. While single infection with any STH did not increase the odds of being anaemic, co-infections exerted a significant influence on the prevalence and severity of anaemia in the cohort. The prevalence of moderate and severe anaemia in participants with co-infections is at least two folds higher than the prevalence in those with single infection of any STH and non-infected individuals. Anaemia as a consequence of co-infections or polyparasitism has been highlighted in previous research [2, 44]. The increased presence of moderate and severe anaemia in participants with co-infections may be attributed to the dual impact of the mechanisms causing anaemia. While malaria may cause anaemia through a combination of haemolysis, increased splenic clearance of infected and uninfected red blood cells, shortened red cell life span and cytokine-induced dyserythropoiesis [45–47], the impact of *Ascaris* infection on anaemia is less clear, although it may influence the nutritional status [48]. Findings from this study revealed no relationship between *Ascaris* or *Trichuris* infections and the Hb level. Nevertheless, the fact that up to 59.5% of individuals negative for all parasite species were anaemic- with some having moderate or severe anaemia- highlights the significant contribution of other factors to anaemia that this study did not investigate.

The Hb, Hct, RBC, WBC and platelet values were significantly lower in individuals with co-infections as compared to single infections with either STH or *P. falciparum.* The similarities in mean red cell indices in those with a single STH infection and lower values in those co-infected with a helminth only or *P. falciparum* single infection accentuate the influence of any co-infection and *P. falciparum* infection on haematological parameters. Although all mean platelet counts were within the normal range (150–400 × 10^9^ L), participants infected with *Trichuris* and co-infection with a STH had significantly higher mean platelet counts than their counterparts. While anecdotal case reports exist on trichuriasis and laboratory values [49, 50], in line with the findings of this study, the platelet counts were within the normal range. Nevertheless, the significant increase in platelet counts warrants further investigation to specifically clarify the role of *Trichuris* leading to such an increase*.* On the other hand, those with co-infections had significantly lower platelet counts that approached the lower limit of normal values. This may be attributed to the corollary of *P. falciparum* on platelet counts as thrombocytopenia has been reported as a common feature of falciparum malaria [51, 52]. Some of the speculated mechanisms leading to thrombocytopenia include: coagulation disturbances, shortened platelet life span in peripheral blood and sequestration in non-splenic areas, bone marrow alterations, immunological destruction due to antiplatelet IgG, oxidative stress and, partly, pseudo-thrombocytopenia due to clumping of platelets [52–54].

Even though the mean WBC counts in participants with single infection of *Ascaris* or *Trichuris* were similar, the prevalence of leucopenia was significantly higher in those infected with *Ascaris* and in those with co-infections of *Ascaris* and *Trichuris*. The low prevalence of helminth infections and leucopenia in the studied population limits the power of this finding. In addition to this limitation, eosinophilia, which is known to occur alongside helminth infection, was not investigated to account for the variation in the observation. Nonetheless, the fact that helminth infections were common in adolescents and the adult population, while eosinophilia has been shown to be less frequent as age increases [55] merits further investigation into the effects of *Ascaris* infection on total white cell and differential counts.

Haematological manifestations such as microcytosis and leucopenia observed in the participants have been linked to *P. falciparum* infection in a previous study [36]. The high occurrence of microcytosis in the two groups with the highest prevalence of anaemia in individuals with co-infections and *P. falciparum* single infection, is not surprising. Microcytosis have been associated with lower Hb levels [56, 57]. However, the occurrence of microcytosis in individuals negative for STH and *P. falciparum* infections and the high prevalence of anaemia in these individuals suggests that an undiagnosed iron deficiency possibly contributes to the occurrence of anaemia [43].

## Conclusions

The findings of this study suggest that STHs, malaria and anaemia are still of public health concern among plantation communities in the Mount Cameroon area. Co-infections negatively influenced haematological values and indices. The tea plantation area, age and lack of access to potable water constitute significant risk factors for acquiring STH infections, while the age groups 15–25 years and 26–35 years, and being female were significant risk factors for acquiring anaemia.

In spite of governmental efforts to control these infections, the presence of infections in groups not targeted during control programmes as well as environmental contamination with helminth ova constitute a barrier to the achievement of the desired goal of reduction or eradication. In addition to the inclusion of other at-risk groups in control programmes, potable water should be provided in plantations and communities, and environmental sanitation needs to be improved.

## Additional file

Additional file 1: Multilingual abstract in the five official working languages of the United Nations. (PDF 748 kb)

## Abbreviations

AMP: Asymptomatic malaria parasitaemia
CDC: Cameroon Development Corporation
*CI*: Confidence interval
CMP: Clinical malaria parasitaemia
epg: Eggs per gram
GMED: Geometric mean egg density
GMPD: Geometric mean parasite density
Hb: Haemoglobin
Hct: Haematocrit
MCH: Mean corpuscular haemoglobin
MCV: Mean corpuscular volume
*OR*: Odds ratio
RBC: Red blood cell
SD: Standard deviation
STH: Soil-transmitted helminth
WBC: White blood cell
WHO: World Health Organization
