# Supplementary material for: Soil-transmitted helminths and plasmodium falciparum malaria among individuals living in different agroecosystems in two rural communities in the mount Cameroon area: a cross-sectional study
Source: Infect Dis Poverty. 2017 Mar 16;6:67. doi: 10.1186/s40249-017-0266-6 (PMC5353792; doi:10.1186/s40249-017-0266-6)
Supplement: Additional file 1: — Multilingual abstract in the five official working languages of the United Nations. (PDF 748 kb) [file 40249_2017_266_MOESM1_ESM.pdf]

Translation of the abstract into the five official working languages of the United Nations

الديدان الطفيلية المنقولة عن طريق التربة وملاريا *المتصورة المنجلية* بين الأفراد الذين يعيشون في المناطق الزراعية المختلفة في اثنين من المجتمعات الريفية في منطقة جبل الكاميرون: دراسة إقليمية

إيرين ول نغول سمبيلي، غلاديس بلانكا نيكمنجي، هيلين كوكو كيمبي

خلاصة

تقدمة: لا تزال تعتبر الديدان الطفيلية التي تنتقل عن طريق التربة (STHs) والتهابات *المتصورة المنجلية* مشكلة للصحة العامة في الكاميرون. أجريت دراسة إقليمية في منطقة "جبل الكاميرون" لتحديد مدى انتشار وشدة STHs وعدوى *المتصورة المنجلية* للأفراد الذين يعيشون في البيئات الزراعية المختلفة، لتقييم تأثير هذه الأمراض على مؤشرات الدم وتحديد الأخطار المرتبطة بعدوى STH.

الأسلوب: تم الكشف عن الطفيليات والملاريا باستخدام طريقة كاتو-كاتز وصبغة جيمزا لشرائح الدم، بالتوالي. تم الحصول على تعداد كامل لخصائص الدم باستخدام عداد الخلايا الالكتروني. تم تحليل عينات التربة باستخدام السكروز بطريقة التعويم والترسيب. تمت مقارنة المتغيرات النوعية والمستمرة على النحو المطلوب في حين تم استخدام نماذج الانحدار اللوجستي لتقييم خطر عدوى STH وفقر الدم.

النتائج: من خلال فحص 450 مشترك، تم تشخيص STHs، والمتصورة المنجلية والتهابات أخرى في 14% 33.3% و 5.6% من المشاركين، على التوالي. معدلات عالية لانتشار *الإسكارس* (18.8%) *والمسلكة* (7.9%) لدى المشاركين من مناطق زراعة الشاي مقارنة مع أولئك من مناطق زارعة الموز والنخيل، مع عوارض متماثلة في كثافة البيض. كما تمت مقارنة انتشار *المتصورة المنجلية* وكثافة الطفيلي بين البيئات الزراعية المختلفة. معدل انتشار فقر الدم 64.2%. انتشار عوارض أمراض الدم معتدلة (48%) خطيرة (8%) وفقر الدم ونقص الكريات البيض (26.9%)، وتقزم الكريات الحمر (30.8%) أعلى بكثير بين المشتركين المصابين *بالمتصورة المنجلية* للمشاركين المصابين. أظهرت عينات من التربة من المزارع معدلات أعلى لانتشار بيض STH مقارنة بعينات التربة من المناطق المحيطة بحفر الصرف الصحي وصنابير المياه العامة. كانت المعدلات للذين يعيشون في منطقة مزارع الشاي (*OR* = 3.07)، العمر (*AOR* = 1.49) وصعوبة الوصول للمياه الجارية (*OR* = 2.25)، حيث تعتبر من العوامل المسببة لالتهابات STH، في حين أن الفئات العمرية بين 15-25 سنة (*OR* = 2.928) وبين 26-35 عاما (*OR* = 2.832)، بين الإناث (*OR* = 2.671) من الفئات المعرضة لفقر الدم.

الاستنتاجات: لاتزال الديدان الطفيلية والملاريا وفقر الدم مصدر قلق للصحة العامة في المجتمعات الزارعية. حيث تساهم في التهابات تؤثر سلبًا على قياسات الدم. قد تحديد بيئة زراعة الشاي والعمر وصعوبة الوصول للمياه الجارية من العوامل المسببة لعدوى STH.

喀麦隆火山周边 2 个农村不同农业生态系统居民的土源性蠕虫和恶性疟原虫感染情况的横断面调查

Irene Ule Ngole Sumbele, Gladys Belanka Nkemnji, Helen Kuokuo Kimbi

摘要：

引言：土源性蠕虫病和恶性疟仍然是喀麦隆的公共卫生问题。在喀麦隆火山周边地区开展了一项横断面研究以掌握不同农业生态系统居民的土源性蠕虫和恶性疟的感染情况和感染强度、评价它们对血液学参数的影响并寻找土源性蠕虫感染的相关危险因素。

方法：采用加藤厚涂片法和血涂片吉姆斯染色法分别检测土源性蠕虫和疟原虫。采用自动血液学分析仪获取完整的血液计量参数。采用蔗糖漂浮沉淀法检测土壤样本。根据要求对分类变量和连续性变量进行比较，采用 logistic 回归分析土源性蠕虫感染和贫血的危险因素。

结果：共有 450 名参加者，其中土源性蠕虫、恶性疟原虫和混合感染分别为 14.0%、33.3% 和 5.6%。茶叶种植区居民的蛔虫和鞭虫感染率分别为 18.8%和 7.9%，显著高于香蕉种植区和棕榈树种植区，但是在虫卵密度方面没有差异。不同农业生态系统的恶性疟的患病率和血液原虫密度类似。总贫血率为 64.2%。中度和重度贫血分别为 48.0%和 8.0%，白细胞减少和小红细胞增多症分别为 26.9%和 30.8%，这些血液学异常在恶性疟和土源性蠕虫共感染人群

更为明显。种植区的土壤样本土源性蠕虫卵显著高于厕所和公共水龙头地区。生活在茶叶种植区、年龄和缺乏可移动水源是土源性蠕虫感染的危险因素，OR 分别是 3.07、1.49 和 2.25；而 15-25 岁年龄组、26-35 岁年龄组和女性是贫血的危险因素，OR 分别是 2.928、2.832 和 2.671。

结论：土源性蠕虫病、疟疾和贫血仍然是种植区重要的公共卫生问题。共感染对血液学参数有负面影响。茶叶种植生态系统、年龄和缺乏可移动水源是土源性蠕虫感染的重要危险因素。

Translated from English version into Chinese by Men-Bao Qian, through

CHINESE CENTER FOR DISEASE CONTROL AND PREVENTION
NATIONAL INSTITUTE OF PARASITIC DISEASES

Helminthes transmis par le sol et paludisme à *Plasmodium falciparum* chez des habitants de différents agroécosystèmes dans deux communautés rurales de la région du Mont Cameroun : une étude transversale

Irene Ule Ngole Sumbele, Gladys Belanka Nkemnji, Helen Kuokuo Kimbi

Résumé

Contexte : Les infestations par les helminthes transmis par le sol (HTS) et l'infection par *Plasmodium falciparum* restent des problèmes de santé publique au Cameroun. Nous avons réalisé une étude transversale dans la région du Mont Cameroun afin de déterminer la prévalence et l'intensité des infections par les HTS et *P. falciparum* chez des sujets vivant dans des agroécosystèmes différents, d'évaluer l'influence de ces infections sur les paramètres hématologiques et d'identifier les facteurs de risque des helminthiases à HTS.

Méthodes : Les HTS ont été détectés à l'aide de la méthode de Kato-Katz et les parasites du paludisme par coloration de Giemsa sur frottis de sang. La numération-formule sanguine des sujets a été obtenue à l'aide d'un analyseur d'hématologie automatique. Des échantillons de sol ont été analysés par la méthode de flottation et sédimentation au sucrose. Les variables catégoriques et continues ont été comparées selon les besoins et les facteurs de risque d'helminthiases à HTS et de paludisme ont été évalués à l'aide de modèles de régression logistique.

Résultats : Des HTS ont été décelés chez 14,0 % des 450 participants examinés, *P. falciparum* chez 33,3 % et des co-infections chez 5,6 % des participants. Des prévalences significativement plus élevées d'infestations par *Ascaris* (18,8 %) et *Trichuris* (7,9 %) ont été observées parmi les participants provenant des zones de théiculture par rapport à ceux des régions de culture de bananiers et de palmiers, avec des tendances similaires dans la densité des œufs. La prévalence de *P. Falciparum* et la densité de parasites étaient du même ordre dans les différents agroécosystèmes. La prévalence globale de l'anémie était de 64,2 %. La prévalence des manifestations hématologiques du paludisme telles qu'une anémie modérée (48,0 %) ou sévère (8,0 %), une leucopénie (26,9 %) ou une microcytose (30,8 %) était significativement plus élevée parmi les participants co-infectés par *Plasmodium* et des HTS. Les échantillons de sol des plantations présentaient les plus fortes prévalences d'œufs de HTS par rapport aux échantillons de sol prélevés autour de latrines et de robinets d'eau publics. Le fait de vivre dans une région de théiculture (*OR* = 3,07), l'âge (*ORA* = 1,49) et l'absence d'accès à l'eau potable (*OR* = 2,25) ont été identifiés comme des facteurs de risque d'helminthiase, tandis que l'appartenance aux groupes d'âge de 15 à 25 ans (*OR* = 2,928) ou 26 à 35 ans (*OR* = 2,832) et au sexe féminin (*OR* = 2,.671) représentait des facteurs de risque d'anémie significatifs.

Conclusions : Les HTS, le paludisme et l'anémie sont encore des préoccupations de santé publique parmi les populations des plantations. Les co-infections ont un effet péjoratif sur les paramètres hématologiques. L'agroécosystème de la théiculture, l'âge et l'absence d'accès à l'eau potable ont été identifiés comme des facteurs de risque significatifs d'helminthiases à HTS.

Translated from English version into French by Suzanne Assenat, through

TRANSLATORS
WITHOUT BORDERS

Геогельминты и Плазмодии тропической малярии среди людей, живущих в различных агроэкосистемах в двух сельских общин в районе горы Камерун: по-секционное исследование

Irene Ule Ngole Sumbele, Gladys Belanka Nkemnji, Helen Kuokuo Kimbi

Реферат

Фон: Геогельминты (STHs) и малярийная Плазмодия инфекции остаются проблемы общественного здравоохранения в Камеруне. В по-секционном исследовании была проведена в районе горы Камерун, чтобы определить распространенность и интенсивность STHs и инфекции P. falciparum у людей, живущих в разных агроэкосистемах, для оценки влияния этих инфекций на гематологические показатели; и чтобы идентифицировать факторы риска, связанные с STH инфекций.

Методы: STH и малярийных паразитов были обнаружены с помощью Като-Кац способ и Giemsa окрашиванием мазока крови, соответственно. Были полными значения анализа крови получены при использовании автоматического анализатора гематологии. Образцы почвы были проанализированы с использованием сахарозы методом седиментации, флотации. Категориальные и непрерывные переменные сравнивались и логистические регрессионные модели были использованы для оценки факторов риска для STH инфекций и анемии.

Результаты: Из 450 обследованных участников, STHs, P. falciparum и смешанной ко-инфекций были обнаружены в 14.0%, 33.3% и 5,6% у участников, соответственно. Значительно более высокая распространенность Аскарид (18.8%) и Власоглавы (7.9%) наблюдались у участников с чайных плантаций местности по сравнению с теми из банана и территории пальмовой плантации, с аналогичными тенденциями в плотности яиц. Распространенность P. falciparum и плотности паразита были сопоставимы между различными агроэкосистем. Общая распространенность анемии была 64.2%. Распространенность гематологических проявлений, таких как умеренная (48.0%) и тяжелой (8.0%) анемия, лейкопения (26.9%) и бронхиальной (30.8%) были достоверно выше среди *Plasmodium*-STH ко-инфицированных участников. Образцов почвы с плантаций показали высокую распространенность STH яйца, по сравнению с образцами почвы с участков вокруг ямы унитазов и водопроводных кранов. Живя в чайной плантации уголок (OR = 3.07), возраст (AOR = 1,49) и отсутствие доступа к питьевой воде (OR = 2.25) были идентифицированы как факторы риска для STH инфекций, в то время как возрастные группы 15-25 лет (OR = 2.928) и 26-35 лет (OR = 2.832), и женщины (OR = 2.671) были значительные факторы риска для анемии.

Заключение: STHs, малярия и анемия по-прежнему являются проблемой общественного здравоохранения в общинах плантации. Co-infections негативно влияют на haematological показатели. Чай сельское хозяйство агроэкосистемы, возраст и отсутствие доступа к

питьевой воде были определены как значимые факторы риска для STH инфекций.

Translated from English version into Russian by Hao-Qi Zhang

Helmintos transmitidos a través del suelo y malaria por *Plasmodium falciparum* en individuos residentes en distintos agroecosistemas en dos comunidades rurales en la zona del Monte Camerún: estudio transversal

Irene Ule Ngole Sumbele, Gladys Belanka Nkemnji, Helen Kuokuo Kimbi

SINOPSIS

Antecedentes: las infecciones causadas por helmintos transmitidos a través del suelo (HTS) y *Plasmodium falciparum* siguen constituyendo problemas de salud pública en Camerún. Se realizó un estudio transversal en la zona de Monte Camerún para establecer la prevalencia e intensidad de las infecciones por HTS y *Plasmodium falciparum* en individuos residentes en distintos agroecosistemas, evaluar la influencia de estas infecciones en parámetros hematológicos e identificar los factores de riesgo asociados a infecciones por HTS.
Metodología: Los parásitos HTS y de malaria se detectaron con la técnica Kato-Katz y la tinción Giemsa, respectivamente. Los valores del hemograma completo se obtuvieron mediante un analizador automático para hematología. Las muestras de terreno se analizaron mediante la técnica de flotación sedimentación de sacarosa. Se compararon las variables categóricas y continuas según fuese necesario y se emplearon modelos de regresión logística para evaluar los factores de riesgo para las infecciones por HTS y la anemia.
Resultados: se detectaron HTS, *P. falciparum* y coinfecciones mixtas en el 14,0%, 33,3% y 5,6%, respectivamente, de los 450 participantes que se analizaron. Se detectaron prevalencias bastante mayores de infecciones de *Ascaris* (18,8%) y *Trichuris* (7,9%) en participantes procedentes de zonas de plantaciones de té en comparación con aquellos de zonas de plantaciones de banana y palmeras. Las tendencias de densidad de huevos eran asimismo similares. La prevalencia y densidad parasitaria de *P. falciparum* eran similares en todos los agroecosistemas. La prevalencia total de anemia fue del 64,2%. La prevalencia de manifestaciones hematológicas como anemia moderada (48,0%) y severa (8,0%), leucopenia (26,9%) y microcitosis (30,8%) fue bastante mayor en participantes con coinfección de *Plasmodium*-HTS. Las muestras del terreno de las plantaciones tenían las mayores prevalencias de huevos de HTS comparadas con las muestras de terreno en las zonas de letrinas de hoyo y grifos de agua públicos. Se identificaron la residencia en una zona de plantación de té (CP=3,07), la edad (CPA=1,49) y la falta de acceso a agua potable (CP=2,25) como factores de riesgo para infecciones HTS. Por otra parte, se identificaron el grupo de edad 15-25 años (CP=2,928) y 26-35 años (CP=2,832) y el sexo femenino (CP=2,671) como importantes factores de riesgo para la anemia.
Conclusiones: los HTS, la malaria y la anemia siguen siendo un problema de salud pública en comunidades situadas en torno a plantaciones. Las coinfecciones tienen una influencia negativa sobre los parámetros hematológicos. Se identificaron el agroecosistema de cultivo de té, la edad y la falta de acceso a agua potable como factores importantes de riesgo para infecciones por HTS.

Translated from English version into Spanish by Sergio Campo, through

TRANSLATORS
WITHOUT BORDERS
